# Virtual Reality in the Neurosciences: Current Practice and Future Directions

**DOI:** 10.3389/fsurg.2021.807195

**Published:** 2022-02-18

**Authors:** Hayden Scott, Connor Griffin, William Coggins, Brooke Elberson, Mohamed Abdeldayem, Tuhin Virmani, Linda J. Larson-Prior, Erika Petersen

**Affiliations:** ^1^College of Medicine, University of Arkansas for Medical Sciences, Little Rock, AR, United States; ^2^Department of Neurosurgery, University of Arkansas for Medical Sciences, Little Rock, AR, United States; ^3^Department of Anesthesiology, University of Arkansas for Medical Sciences, Little Rock, AR, United States; ^4^Department of Neurology, University of Arkansas for Medical Sciences, Little Rock, AR, United States; ^5^Department of Biomedical Informatics, University of Arkansas for Medical Sciences, Little Rock, AR, United States; ^6^Department of Pediatrics, University of Arkansas for Medical Sciences, Little Rock, AR, United States; ^7^Department of Psychiatry, University of Arkansas for Medical Sciences, Little Rock, AR, United States; ^8^Department of Neurobiology and Developmental Sciences, University of Arkansas for Medical Sciences, Little Rock, AR, United States

**Keywords:** virtual reality, augmented reality, neurosurgery, artificial intelligence, neuroscience

## Abstract

Virtual reality has made numerous advancements in recent years and is used with increasing frequency for education, diversion, and distraction. Beginning several years ago as a device that produced an image with only a few pixels, virtual reality is now able to generate detailed, three-dimensional, and interactive images. Furthermore, these images can be used to provide quantitative data when acting as a simulator or a rehabilitation device. In this article, we aim to draw attention to these areas, as well as highlight the current settings in which virtual reality (VR) is being actively studied and implemented within the field of neurosurgery and the neurosciences. Additionally, we discuss the current limitations of the applications of virtual reality within various settings. This article includes areas in which virtual reality has been used in applications both inside and outside of the operating room, such as pain control, patient education and counseling, and rehabilitation. Virtual reality's utility in neurosurgery and the neurosciences is widely growing, and its use is quickly becoming an integral part of patient care, surgical training, operative planning, navigation, and rehabilitation.

## Introduction

In 1939 the view-master, a simple non-electric handheld device that created a three-dimensional environment from a slide, became the first patented virtual reality device in the United States (1). Today, virtual reality (VR) has progressed from the 1900s simplistic slide display to an innovative wearable device that generates a three-dimensional environment able to be manipulated by the user in a seemingly real or physical way (2, 3). As such, VR has expanded into a multibillion-dollar industry led by technology giants such as Facebook, Sony, and Microsoft (4). Though it has primarily been used for entertainment, the implementation of VR in medicine has been explored for the past 25 years (1, 4). Specifically within neurological surgery and the neurosciences, VR has been widely applied to improve peri-operative planning, surgical training, and rehabilitation (2, 3).

While VR systems such as the Oculus Quest 2 (Facebook Inc., USA) and the HTC VIVE (HTC, Taiwan) produce an immersive environment simulated by a head-mounted display (HMD), augmented reality (AR) involves the superimposition of elements from virtual reality to the real-world environment in the form of holograms or videos (2, 3). We will focus this review on VR applications. Haptic devices provide proprioceptive, vestibular, kinesthetic, or tactile sensory information from the simulated environment to the user, and additionally are often paired with VR devices to increase their utility and provide further user immersion (5). Alongside advances in the combined use of VR with haptic devices, a multitude of different software has also been created to specialize in surgical planning, training, and rehabilitation (5). Our review aims to encompass the past, present, and future applications of VR devices in the field of neurosurgery and the neurosciences regarding medical education, skills training, patient counseling, pain management, rehabilitation, and management of neuropsychiatric disorders. To conclude, we will discuss the obstacles and limitations necessary to successfully unlock the potential of further implementing the use of virtual reality within the field of neurosurgery and the neurosciences.

## Methods

**Figure 1:**
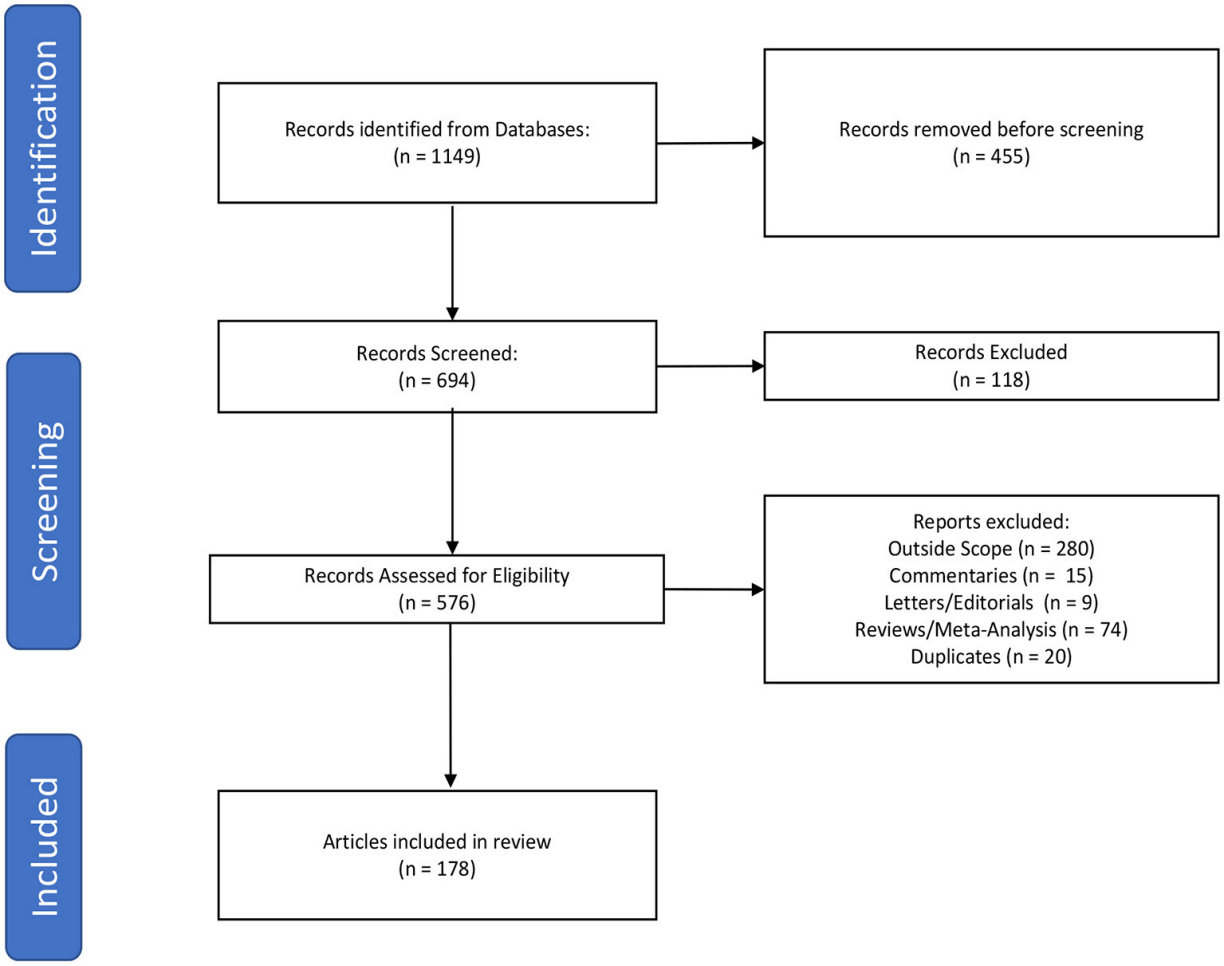
Flowchart demonstrating identification, screening, and inclusion of articles.

The primary literature search was performed by querying the PubMed database to identify literature published addressing the implementation of virtual reality in neurosurgery and the neurosciences. In order to search both MeSH terms and words utilized in the abstract/text, PubMed was queried using “Neurosurgery” OR “Neurosurgical Procedures” OR “Neurosciences” OR “neurosurgery OR neuroscience OR neurosurgical” AND “Virtual Reality” OR “virtual reality” with an English language only and 2010–2021 date restriction. We subsequently performed a secondary search to include the term “augmented reality” in order to fully encompass the scope of our review. Studies that evaluated the use of virtual reality in neurosurgery or the neurosciences were included in this review. Three authors (HS, CG, and WC) independently screened the articles from the literature search and created a list of studies meeting the inclusion and exclusion criteria. Articles were excluded if they were duplicates, systematic reviews, meta-analyses, commentaries, letters to the editor, or outside the scope of the topic in question (Figure 1). Articles were categorized by field and applications of virtual reality (Table 1). The list was reviewed for discrepancies by all parties and any discrepancies were settled by discussion.

**Table 1:** Breakdown of the number of articles referenced in each section.

**Topic**	**Number of selected articles**
Background	5
Neurosurgical training	51
Patient education/counseling	2
Distraction for awake surgery	2
Beyond the OR	21
Pain management	10
Neuropsychiatric conditions	39
Rehabilitation indications	56
Discussion	8

*Some articles are referenced in multiple sections*.

## Applications of VR as a Neurosurgical Skills Training Tool

To initiate our discussion regarding VR in neurosurgery and the neurosciences, it is fundamental to discuss VR's use as an integral teaching supplement and powerful means for simulating procedures. Due to the complex bony architecture of the skull, the application of VR in neurosurgical education has garnered a large amount of attention to aid in developing a comprehensive understanding of neuroanatomy and in practicing surgical skills (2, 5–12). VR models have been shown to be comparable to a cadaver and an atlas in teaching skull-based anatomy (11). Additionally, these models provide reliable localization of key anatomical landmarks in teaching craniofacial trauma when compared to computed tomography (CT) scans and can accurately simulate anatomical features of such structures as the suboccipital vertebral arteries and their surrounding bony architecture in comparison to cadaveric heads (13, 14). Furthermore, VR has been successfully employed to generate three-dimensional models of the sphenoid sinus (15), the clivus (16), and temporal bone (17) for teaching complex neuroanatomy. While the teaching applications involved virtual models, VR has also been utilized as a teaching resource through 360-degree videos of surgical approaches and neurological pathologies (18–22). These 360-degree video procedures include: combined petrosectomy (18), arteriovenous malformation resection (19), structural changes in the brain of a person with corticobasal syndrome, (20) expanded endoscopic endonasal transtuberculum approach for tuberculum sellae meningioma (21), and immersive tutorials in specialized areas such as trauma training (22). These 360-degree videos provide a unique resource for medical students and residents to prepare for, and review, specific cases and enhance their operative education.

The ability of VR to augment traditional teaching methods is increasingly important considering the decreased supply and increased costs of cadaveric specimens (23, 24). The cost-benefit ratio of a VR system that can not only be used multiple times compared to a time-limited cadaver, but also can be used by students around the world, demonstrating benefits of VR. Furthermore, VR has the potential to supplement traditional training methods in the acquisition of fine motor skills for microsurgical procedures (23–28). Neurosurgical simulators in ventriculostomy have already been shown to compliment neurosurgical resident education, Cohen et al. (27), Hooten et al. (29), Schirmer et al. (30), and Haji et al. (31) and include patient-specific scenarios for an endoscopic third ventriculostomy (25). In the realm of spine surgery, VR simulators have been utilized for training in multiple varied surgeries and approaches (32–35). In one study, two groups of senior medical students placed pedicle screws in lumbar sawbones models. One group was trained using the ImmersiveTouch simulator (ImmersiveTouch, USA) and the control group underwent traditional visual and verbal instruction. The students who were trained with a VR simulator outperformed the control group in all measures (35). Although the sample size in this study was very small (26 students were divided into two groups), despite the study population not having as advanced training as practicing neurosurgery residents or attending physicians, potential benefits of training with VR were found. VR simulators have also been applied to training in tumor resection (36, 37), endoscopic nasal surgeries (28, 38), and cerebral aneurysm clipping (39–45). Notably, with the decreased number of cerebral aneurysms treated surgically, VR simulators serve as a valuable tool for early residents to practice specific approaches and techniques and improve their procedure time prior to entering the operating room (39–43, 45). Overall, the implementation of simulators as a foundational part of neurosurgical training contributes to an improvement in psychomotor surgical skills, safety, and cost for neurosurgical training (9, 46, 47).

While VR simulators provide enhanced neurosurgical resident training, simulations also create large data sets (48). The data may then be analyzed to quantify psychomotor skills in neurosurgical training (48–54). When paired with artificial intelligence (AI), links are created between medicine, computer science, and education that can collaboratively revolutionize surgical training (52). The utility of AI to assess psychomotor skills is still formative but has been shown to classify individuals into different expertise levels with an accuracy of over 90% (48–52). By providing a new tool to classify surgical skills training, a shift in the longstanding paradigm of case volume being correlated with skill level could revolutionize the means in which residents are trained. With further improvements in devices and computer science, psychomotor assessment through VR simulators may become an integral component of neurosurgical education.

## Applications of VR In Neurosurgical Patient Education And As A Counseling Tool

Patient education is a cornerstone of medicine. Abiding by the principle of autonomy, each patient should be made fully aware of their medical conditions and understand the available treatment options. Oftentimes neurosurgical procedures are some of the most detailed and complex within surgery, resulting in a large knowledge gap between patient and provider. In an attempt to remedy this gap, some providers have implemented VR technology to educate and counsel their patients on treatment options. For example, Perin et al. (55) showed the educational benefits of VR for patients undergoing surgical removal of intracranial tumors. Patients who underwent an immersive three-dimensional informed consent process involving two surgical planners displayed a higher level of objective comprehension when compared to the patient control group where patients underwent an informed consent process supported by traditional 2D radiological images (55).

Outside of the operating room, VR has also been implemented for patient education and counseling in a clinical setting by engaging patients in interventions that promote overall well-being through behavioral reinforcement and individualized educational approaches (56). The increasing number of individuals affected by chronic health conditions such as addiction, obesity, and diabetes make it imperative to provide patient education that allows individuals to observe their behaviors and review the steps required to better control these conditions. Discussing the potential changes with these patients through a non-judgmental means allows for the development and continuation of the fiduciary patient-physician relationship.

## Applications of VR For Immersive Distraction During Awake Surgery

Surgical treatment can be emotionally challenging for patients, especially patients that require neurosurgical intervention. Requiring brain or spine surgery commonly represents a life-altering event that oftentimes can lead to anxiety and distress in patients. A sincere surgeon-patient relationship remains crucial when patients are experiencing these intimidating situations. It is imperative to ensure that each patient has a full understanding of the recommended procedure. Surgeries are increasingly performed under monitored anesthesia care or, more rarely, without anesthesia other than Tylenol to reduce the chance of post-operative deficit or to improve the likelihood of a successful surgical outcome, such as precise position of a spinal cord stimulator. For example, awake craniotomies are commonly performed for patients that are affected by intractable epilepsy as well as patients undergoing tumor removal. During these procedures, the patient must be conscious during critical portions of the operation so that the surgeon remains able to monitor functions controlled by eloquent areas of the brain in close proximity to the operative field (57). VR technology has been implemented as a way to immerse patients in a simulated environment that mimics the operating room during neurosurgical procedures in order for them to develop and practice coping mechanisms (58). Additionally, there is potential to parallel this idea by using VR to immerse patients in simulated environments that distract them from the physical environment of the operating room while undergoing neurosurgical procedures.

## Applications of VR In The Operating Room And Beyond

The same advantages that VR provides to surgical education in its ability to recreate three-dimensional anatomically precise models make it attractive for preoperational planning in neurosurgery. For example, as opposed to traditional two-dimensional magnetic resonance imaging (MRI) and computer tomography (CT) images, three-dimensional images created using virtual reality enabled neurosurgeons to study tumor-related anatomy more effectively in anterior skull base and parasagittal meningioma surgeries (21, 59–62). Virtual reality-driven 3D reconstruction navigation has also shown improvements over traditional image modalities in craniofacial, sellar, and infratentorial tumor resection, as well as temporomandibular joint arthroplasty surgeries (63–66). Additionally, virtual reality models provide the capability to describe orientation and shapes in which traditional imaging techniques lacked, for example areas used in microsurgical approaches such as the foramen of Monro (67).

Virtual reality images have also been shown to improve cerebrovascular surgeries by improving aneurysm detection, surgical planning, and the ability to simulate the procedure in virtual reality (68, 69). With regards to trauma, virtual reality appears to be as accurate as neuronavigation in planning minimally invasive cranial procedures (70). Virtual reality also shows excellent potential in planning approaches to deep brain structures such as the third ventricle and improved freehand external ventricle drain placement (71, 72).

Virtual reality provides a unique precise surgical model that allows the surgeon to simulate and detect structures such as a fistula in spontaneous cerebrospinal otorrhea, which traditional imaging modalities failed to identify (73). VR has been used to visualize the associated vasculature prior to hemangioblastoma resection at the craniocervical junction and is useful in planning microvascular decompression at the cerebellopontine angle (74, 75). Furthermore, virtual reality has been shown to improve surgical planning of minimally invasive spine surgeries such as spinal decompression and fusion with comparable accuracy and far less intra-operative radiation (76, 77). In addition, virtual reality is also being used as an imaging modality in the evaluation of post-surgical results in procedures such as monosegmental cervical fusion instead of traditional imaging modalities by measuring the smallest cross-sectional area of the intervertebral neuroforamen in the lateral resection region (78). Altogether, these areas highlight the potential for VR being utilized in every stage of an operation, including its surgical planning, surgical guidance, patient anxiety relief, and evaluation of post-surgical results.

## Applications of VR For Acute And Chronic Pain Management

VR technology has been implemented in many scenarios involving the assessment and management of patients with acute and chronic pain (79). Similar to the VR application for distraction during awake neurosurgery, VR has been applied to create a distracting virtual environment for patients with acute pain. For example, when acting as a supplement to standard analgesic therapy, VR distraction decreased subjective pain ratings for worst pain intensity, pain unpleasantness, and time spent thinking about pain (80). Another study involving virtual waiting environments for pain distraction in patients with chronic migraines discussed the effect of VR on laser evoked potential (LEP) vertex and laser-pain ratings in patients (81). The study demonstrates that patients immersed within an ideal virtual waiting area had significantly decreased subjective pain ratings compared to those who had a virtual waiting area depicting a typical hospital waiting environment. However, a study using VR for patients with chronic neck and back pain showed no significant difference in pain intensity in individuals using VR when compared to patients carrying out physical neck exercises (82, 83). The lack of significant results in the latter study could be due the study being under-powered due to loss of participants to follow up. These studies lay a groundwork for future studies to build upon in order to create immersive virtual environments that successfully provide pain relief for patients in the acute and chronic settings by selecting appropriate sample sizes and properly matched controls to minimize confounders (84, 85).

In addition to the management of acute pain, studies have investigated the use of VR in patients with chronic back pain. In order to assess movement induced pain, electroencephalography (EEG) has been combined with VR to assess cortical activity identifying putative mechanisms, such as diminished disinhibition in prefrontal motor areas (86). Identifying the source of the pain could potentially allow for the development of more precise targets when developing pain management plans (86). One study involving patients with spinal cord injury who had chronic pain showed that VR technology provided an analgesic effect by applying asynchronous or synchronous visuotactile stimulation to a patient's back and to virtual legs on a VR display (87). Other applications in patients with chronic pain include the use of VR for the relief of neuropathic pain associated with phantom limb syndrome (88). In addition to chronic pain, conditions originating from a psychological foundation have also been targeted by applications of VR technology. Some of these conditions, including those centered around an individual's perception of their own body image, are discussed in further detail elsewhere in this paper.

## Applications of VR For Neuropsychiatric Conditions

The application of VR and simulated environments has been used in the treatment of patients who have been affected by various neuropsychiatric conditions including specific and situational phobias, schizophrenia (44, 89–91), post-traumatic stress disorder, obsessive-compulsive disorder (92–95), and autism spectrum disorder (96–103). VR has been used in those who experience situational anxiety, such as a fear of speaking in public, in order to create a simulated environment to assist individuals in overcoming the stress of that particular environment. In the case of specific phobias, desensitization and flooding have been commonly used methods that assist individuals overcome their fears (102, 104–109). In desensitization, patients are gradually exposed to items and scenarios that provoke a sense of fear, often beginning with a scenario as simple as imagining the particular fear. With gradual exposure, the goal is for the individual to essentially develop a tolerance to the stimulus to the point where it no longer elicits a sense of fear. On the contrary, flooding involves rapidly exposing individuals to objects or scenario to provoke a fear response in a controlled environment. The application of VR in standardized settings allows providers and patients to create a simulated environment that involves a particular fear-provoking stimulus in hopes of eliminating the fear response, resulting in eventual extinction (102, 104–109).

## Applications of VR for Rehabilitation Indications

Virtual reality has been used extensively to aid in the rehabilitation of neurocognitive diseases, traumatic injuries, and cerebrovascular disorders due to its ability to recreate an immersive, interactive environment with real-time feedback. One unique area that VR is being applied is in the treatment of spatial neglect (110, 111). Prior to VR, one way in which spatial neglect is being treated is using prism adaptation, a system in which a mismatch occurs between the perceived position of a target and its actual position by shifting the field of vision through prismatic googles (110, 111). VR can replicate this process and provide enhanced blinding due to its manipulatable immersive environment (110, 111). VR's application in this area is of interest since the results of prism adaptation have been questionable at best (112). Nevertheless, other forms of rehabilitation for chronic neglect patients such as VR visual scanning rehabilitation programs, games in which patients actively pay attention to and interact to stimuli on their affected side, seems to induce a plasticity process that benefits chronic neglect patients (113, 114).

VR feedback has been paired with a gravity-compensating multi-joint exoskeleton for the upper extremities to facilitate and provide feedback in reach-to-grasp rehabilitation in stroke patients (115–117). Specifically, VR environments provide quantitative assessment of upper and lower limb movements, that can be utilized to adapt subsequent sessions thereby aiding in stroke rehabilitation (118). A randomized-controlled trial suggests that the addition of VR feedback to traditional rehabilitation of the upper limb promotes better stroke outcomes regardless of stroke etiology (119). VR walking simulators further show potential in aiding stroke patients for lower extremity rehabilitation (120). Given the potential application VR has for upper and lower extremity rehabilitation, VR rehabilitation systems grounded in neuroscience are being created to assist in the recovery of motor functions after neurologic insult (121, 122).

One aspect of stroke rehabilitation involves the use of multisensory modalities to improve our ability to detect and discriminate stimuli and create an optimal environment for learning (123). VR provides the ability to recreate these environments through simple tactile feedback games. Moreover, a study that combined VR with levodopa for acute stroke rehabilitation has been tested to evaluate its synergistic neuroplastic effect. While the study has limited power (*n* = 8), it did succeed in showing significant improvements in upper extremity kinematic function in the VR/levodopa group in comparison to the control group receiving levodopa alone (124). Other approaches paired with VR to generate this multisensory effect include the Michelangelo effect, a term coined by a study evaluating the impact of a VR program where the user creates a masterpiece when painting on a virtual canvas (125).

Because rehabilitation for chronic stroke patients continues long after the time spent in rehabilitation facilities, interest has been growing into creating VR programs for consumer head-mounted displays (126, 127). More primitive non-immersive virtual reality systems such as the Nintendo Wii (Nintendo, Japan) have already been shown to provide sustained benefit to stroke patients (128, 129). As a result, the rehabilitation gaming systems (RGS) model of stroke rehabilitation has been suggested to be more effective than traditional stroke therapies (130). Additionally, VR games for post-stroke treatment appear to be beneficial in treating stroke sequelae by improving the symmetry of body temperature, balance, and functionality of stroke patients (131).

When combined with robot-assisted gait training, VR seems to be a viable alternative to traditional motor rehabilitation in patients with multiple sclerosis (MS) (132). VR's potential to use dual-task therapy for multiple sclerosis patients when paired with a treadmill is currently being studied (133). Furthermore, VR games have been used as motor tasks in MS patients to evaluate the effect of cognitive function and fatigue on motor performance improvement (134). Taken together, these studies highlight the potential role of VR in MS rehabilitation.

For spinal cord injuries, VR games have gained interest in their application for balance training. However, in one study, semi-immersive VR therapy when paired with conventional rehabilitation as opposed to traditional rehabilitation alone revealed no significant differences (135). VR does show promise in its ability to induce illusionary spinal movements that eclipse standard mirror therapy for the treatment of limb pain by amplifying small neck movements into perceived larger ones (136). VR has gained interest in treating Alzheimer's disease by inducing neuroplasticity (137) and providing gait training (138) through the pairing of multiple daily life task simulators with walking on a treadmill. Additionally, VR is being incorporated into moving platform systems to improve a patient's gait and posture (139). While VR with moving tasks in elderly patients, such as a walking on a treadmill, appears dangerous, a study suggested that for balance in the elderly these systems appear to provide the most benefit for safety and outcomes when combined with a mixed exercise (140). VR has warranted attention in the area of rehabilitation in people who experienced a traumatic brain injury. Specifically, driving VR interfaces have shown the benefits of cognitive rehabilitation of working memory to increase efficiency in neural networks (141, 142).

Virtual reality is also being actively used in the rehabilitation of developmental and ocular neurological diseases. For instance, VR coupled with robotic exoskeleton devices has been used to assess the motor deficits in children with fetal alcohol syndrome (143). Due to the ability of head-mounted displayed VR systems to manipulate each eye, VR systems have been developed to induce neuroplasticity in children with amblyopia (144). Within this same field of rehabilitation, a VR system involving squashing bugs was developed to rehabilitate adults suffering from loss of stereo vision, the ability to process the depth of an image using binocular vision, from conditions such as strabismus or amblyopia (145). This is of significance due to the limited number of studies documenting recovery of stereo vision in adults as opposed to children, which may pave the way for an exciting new area in rehabilitation using VR (145).

VR's ability to provide haptic feedback through reach and grasp movements in a VR environment has gained interest in rehabilitating children with mild unilateral and dystonic cerebral palsy (146, 147). Additionally, studies are now being designed to evaluate the combination of motor movement training through VR paired with anodal transcranial stimulation, a form of transcranial stimulation that favors cortical excitability and depolarization, in the rehabilitation of upper limb movement in children with Down syndrome (148). VR with anodal transcranial stimulation also shows promising results for improving gait in people with motor disorders such as cerebral palsy (149). VR programs have been used to test spatial learning and memory in surgery-naive temporal lobe epilepsy, which suggested the integrity of both hemispheres is critical for spatial learning and memory (150).

In the rehabilitation of Parkinson's disease patients, VR facilitated motor-imitation therapy has been shown to have many applications [for a detailed recent review see (151)]. Studies have shown VR to help reduce PD patient's hypometria (152, 153). Finger tapping testing for Parkinson's patients in a virtual environment appears to yield similar results compared to standard finger tapping testing (154) VR combined with treadmill rehabilitation shows some feasibility in improving gait by modifying brain activation patterns using optic flow, the experienced change in scenery in virtual reality by walking (155–158). To evaluate Parkinson's patients with freezing of gait (FOG), VR has been employed and suggests that Parkinson's patients with FOG show slower motor initiation, increased movement hesitation, and marked impairment of motor movement inhibition (159, 160). Thus, VR programs for Parkinson's patients have been created to improve movement and problem solving by completing tasks in a virtual environment (161). When paired with the Nintendo Wii, exercise appears to produce a more significant therapeutic effect for Parkinson's patients than conventional exercise alone (162). VR rehabilitation programs seem to be as effective as traditional rehabilitation yet can better determine the overall improvement due to data input from the device (163).

## Discussion

The rapid advancements in technology, including those involving VR devices, have led to complex technology that can only be used to their full potential if the operator has a foundational understanding of the technology being implemented (164). Once that underlying issue is overcome, the application of virtual reality poses several other obstacles that can complicate their use in the neurosciences. For example, many neurosurgical procedures require intraoperative imaging, such as MRI or CT; however, the standard virtual reality devices controllers and HMDs are not compatible with MRI and implementation in such settings would require this development (165). As previously mentioned, intractable epilepsy is a common indication for neurosurgical care; however, the possibility of photosensitive seizures induced by VR devices has been discussed in the literature. Although the medical literature did not support the idea of VR resulting in photosensitive seizures, many HMD manufactures, such as Oculus, include risk of seizure in their health and safety warnings with use of their product (166). In a small study of patients with Parkinson's Disease, the use of VR has been associated with the development of visual hallucinations (167). Unwanted effects from VR immersive systems may be specific to the individuals it is used for, and a system may need to be designed according to its particular use. Another study reported participants experienced unpleasant symptoms, including nausea and disorientation, related to virtual reality sickness and effects (VRISE) (168). The maximum duration of VR immersion should be limited to between 55 and 70 min, as longer exposure increases the probability of the occurrence and intensity of VRISE (169). VR has been adapted for use in specific medical scenarios, but it is important to highlight that patients and not the medical providers are the end users (170). Therefore, implementing the use of VR in the neurosciences should not be solely focused on the advantage to the medical providers. For the long-run, VR technology needs to be implemented in ways that are mutually beneficial to patients and physicians, thereby enhancing the patient-physician relationship. We have provided a table summarizing our findings related to the application of VR both inside and outside the operating room (Table 2). Many of these applications are novel, as the technology itself is relatively novel, and delineation of proper guidelines is ongoing.

**Table 2:** Summary table of findings and pathologies discussed in the referenced articles.

**Topic**	**Reference**	**Findings**
**Neurosurgical skills and training tools**		
Anatomy education	(2, 5–17)	(11) Anatomy teaching comparable to cadaver/atlas
360-degree education video	(18–22)	
VR simulators	(23–47)	(23) Apparent cost benefit of simulation training (24) Apparent cost benefit of simulation training (35) Simulation group outperformed control group
Artificial intelligence with VR	(48–54)	
Patient education/counseling	(55, 56)	(55) Increased comprehension in VR group over control group
VR during awake surgery	(57, 58)	
VR in the operating room and beyond		
VR imaging compared to traditional imaging	(21, 59–62)	
3D navigation using VR	(63–67)	
VR for operation planning	(68–78)	(70) Suggests VR accuracy is equal to that of neuronavigation In planning minimally invasive procedures (76) VR utility in surgical planning and post-surgical evaluation in spinal decompression (77) VR utility as an image modality in spinal fusion
**Acute/chronic pain management**		
Acute Pain	(79–85)	(80): VR's utility as a supplement to standard anesthesia decreased subjective pain rating (82) Study suggested VR did not improve chronic back pain rating
Chronic Pain	(86–88)	
**Neuropsychiatric conditions**		
Schizophrenia	(44, 89–91)	
PTSD and OCD	(92–95)	
Autism	(96–103)	
Desensitization/Flooding	(102, 104–111)	
**Rehabilitation**		
Spatial neglect	(112–116)	(113): VR rehabilitation induces neuroplasticity over traditional rehabilitation (114) fMRI study illustrating VR's possibility in inducing neuroplasticity for chronic neglect patients
Stroke	(117–131)	(119) Highlights the benefits of adding VR rehabilitation in addition to traditional rehabilitation (124) Highlights the benefit of VR with pharmacology for stroke rehabilitation
Multiple Sclerosis	(132–134)	
Spinal cord injuries	(135, 136)	(135) VR paired with conventional spine therapy showed no significant improvement
Alzheimer's disease	(137)	
Gait training	(138–140)	
Traumatic brain injury	(141, 142)	
Fetal alcohol syndrome	(143)	
Amblyopia	(144, 145)	
Pediatric	(143, 144, 146–149)	
Epilepsy	(150)	
Parkinson's disease	(151–163)	(152) VR utility in imitation training (153) VR utility in imitation training (162) Older study suggesting Wii exercise provided greater therapeutic effect than conventional exercise
**Discussion**		
Competence in VR	(164)	
Incompatibility of VR with standard equipment	(165)	
Risks of using VR	(166–170)	
Prospective view of VR	(171)	

Despite the challenges ahead, VR has become a new tool widely employed across the neurosciences and neurological surgery. From rehabilitation to resident education and acute pain to intra-operative feedback, VR has integrated itself widely throughout many different specialties. As the technology surrounding virtual environments advances and the ability to incorporate haptic feedback provides further immersion to an augmented environment and feedback from said environment, VR will continue to redefine neurosurgical training and patient care. By providing a safe, superior, flexible, and readily available tool, VR has the potential to further shape the future of the neurosciences and neurosurgery. Significant advancements in neurosurgery occur every 20 years, but rapid progressions in technology can assist in transforming the field at a more rapid rate (171). Recently, *Neurosurgical Focus* dedicated an issue solely to virtual and augmented reality, demonstrating the impact of this technology within the field of neurosurgery. Similar to the articles we have discussed in our review, articles within this issue of *Neurosurgical Focus* also highlight how the implementation of virtual and augmented reality have and will continue to result in rapid advancements in operative planning, intraoperative navigation, and neurosurgical training.

## Author Contributions

EP, WC, BE, and MA: main concept and manuscript outline. HS, CG, and WC: literature search, article screening, manuscript draft, and revision. LL-P, EP, WC, TV, BE, and MA: draft edits and commentaries. All authors reviewed article prior to submission.

## Conflict of Interest

The authors declare that the research was conducted in the absence of any commercial or financial relationships that could be construed as a potential conflict of interest.

## Publisher's Note

All claims expressed in this article are solely those of the authors and do not necessarily represent those of their affiliated organizations, or those of the publisher, the editors and the reviewers. Any product that may be evaluated in this article, or claim that may be made by its manufacturer, is not guaranteed or endorsed by the publisher.
